# The Knowledge and Awareness of Parents Regarding Pediatric Obstructive Sleep Apnea in the Central Region of Saudi Arabia

**DOI:** 10.3390/healthcare13090968

**Published:** 2025-04-23

**Authors:** Khalid M. Alkhalifah, Farah Saleh Allabun, Abdulrahman Ahmed Alsughayyir, Waleed Obaid Alharbi, Sarah Abdulaziz Almagushi, Meshal S. Alwabel, Waleed Alhazmi

**Affiliations:** 1 Al-Rass General Hospital, Qassim Health Cluster, Al-Rass 58882, Saudi Arabia; 2 King Saud Hospital, Qassim Health Cluster, Unayzah 56437, Saudi Arabia; 3 College of Medicine, Qassim University, Buraidah 52571, Saudi Arabia; 4 Department of Psychiatry, King Fahad University Hospital, Imam Abdulrahman Bin Faisal University, Al-Khobar 31441, Saudi Arabia; 5 Department of Otolaryngology-Head and Neck Surgery, College of Medicine, Qassim University, Buraidah 52571, Saudi Arabia; w.hazmi@qu.edu.sa

**Keywords:** pediatric OSA, children, awareness

## Abstract

**Background/Objectives**: Pediatric obstructive sleep apnea (POSA) is a long-term sleep disorder characterized by repeated interruptions in breathing during sleep among children. These interruptions result from blockages in the upper airways, causing decreased oxygen intake and disturbed sleep. Assessing parents’ awareness level and the factors affecting their knowledge is vital for enhancing early diagnosis and management of POSA. **Methods**: This was a cross-sectional study that utilized data from a sample of 838 parents in the Central Region of the Kingdom of Saudi Arabia. The participants completed self-administered online surveys, which ensured anonymity. **Results**: Only 320 (38.2%) of the parents demonstrated good knowledge about POSA, while the majority, 518 (61.8%), had poor knowledge. Nearly a third, 261 (31.2%), of the parents indicated that their primary sources of information on POSA were the internet and social media platforms. The prevalence of OSA among children was quite significant, with nearly a quarter, 236 (28.2%), of parents having a child affected by the condition. Commonly recognized symptoms included snoring, reported by 425 (50.7%), and mouth breathing, reported by 156 (18.6%). Frequently cited risk factors included obesity, mentioned by 373 (44.5%), and enlarged tonsils, mentioned by 175 (20.9%). A statistically significant association was found between age, gender, marital status, education level, specialization/work, source of knowledge about POSA, knowing someone with OSA, and having a child with OSA and the level of knowledge about POSA (*p* < 0.05). The study identified several significant factors predicting poor parental awareness of pediatric obstructive sleep apnea (POSA) including gender [AOR = 1.65; 95% CI = 1.220–2.223; *p* = 0.001], source of knowledge about pediatric obstructive sleep apnea [AOR = 1.35; 95% CI = 1.167–1.572; *p* < 0.001], and knowing someone with OSA [AOR = 1.92; 95% CI = 1.301–2.832; *p* = 0.001]. **Conclusions**: The study revealed that parents in the Central Region had limited knowledge about POSA. There were notable gaps in recognizing and understanding the symptoms of POSA and its impact on children’s mental health and academic performance. This underscores the importance of introducing targeted educational programs and initiatives for both parents and healthcare providers to enhance children’s mental health and overall well-being.

## 1. Introduction

Pediatric obstructive sleep apnea (POSA) is a relatively common disorder in children, defined by interrupted breathing during sleep caused by partial or complete blockage of the upper airway. This occurs when the muscles supporting the airway relax, especially during inhalation, leading to airway collapse while the child sleeps [1]. In addition to loud snoring, choking, or gasping for air, obstructive sleep apnea (OSA) leads to fragmented sleep and frequent nighttime awakenings. This disrupted sleep can result in daytime fatigue, morning headaches, and cognitive impairment. In pediatric patients, OSA may also manifest as poor school performance, behavioral problems, and hyperactivity [2].

POSA affects approximately 1–13% of children, with this prevalence varying based on factors such as sex, age, and the presence of other conditions like craniofacial abnormalities or obesity [3,4]. Moreover, obesity and adenotonsillar hypertrophy, which peaks between the ages of 3 and 6, are also shown in another study to be the common risk factors of OSA in children [4,5]. Obstructive sleep apnea symptoms include excessive daytime sleepiness, restless sleep, snoring, inattention, and behavioral issues like hyperactivity and impulsiveness, occasionally leading to attention deficit hyperactivity disorder [6].

POSA’s increasing prevalence is gradually emerging as an international health epidemic, mainly because of the current worldwide epidemic of obesity [7]. Despite its widespread prevalence, POSA is a risky disease that is usually neglected. Although OSA does not pose a direct threat to life, if left untreated, it can cause adverse health effects and complications, including diabetes mellitus, stroke, hypertension, daytime hypersomnia, and cardiovascular diseases [8]. Even though OSA is relatively common, most children probably do not receive a diagnosis [9]. This suggests that a sizable portion of children with OSA remain undiagnosed and untreated by their parents. Ignoring OSA symptoms can have irreversible effects, particularly in children.

In Saudi Arabia, research on POSA is scarce, and the available studies indicate that awareness of the condition needs to be higher among the general public. The study conducted in Jeddah by Alosaimi et al. revealed a need for more knowledge about POSA among the 146 parents surveyed [10]. The study by Alshehri et al. in the Asir region uncovered a general lack of awareness regarding various aspects of POSA among the population [11]. Additionally, research by Saleem et al. among primary care physicians in Saudi Arabia highlighted a need for more knowledge related to sleep medicine [12].

However, no previous studies have specifically examined parental knowledge and awareness of pediatric OSA in Saudi Arabia’s Central Region. The existing knowledge gap highlights the need to bridge the gaps by conducting this study in the Central Region. Therefore, this study sought to assess the knowledge and awareness of parents in the Central Region regarding POSA.

## 2. Materials and Methods

The study adopted a cross-sectional design and was conducted between August 2024 and September 2024 in the Central Region of Saudi Arabia. The inclusion criteria included parents aged 18 years and above, both male and female, who resided in the Central Region. Conversely, the exclusion criteria disqualified individuals under 18 years of age, those without children, and those living outside the Central Region.

The sample size was determined using the Cochrane formula, with a 95% confidence level and a margin of error of 5%. The minimum acceptable sample size was 384, but we adopted a sample of 396 respondents. The study adopted a non-probability convenient sampling technique by including all those who meet the inclusion criteria.

After obtaining permission, we used a validated questionnaire from a previously published article [10]. The pilot study of the questionnaire was tested involving 10% of the target population to see whether the data collection techniques were correct before beginning the study. The participants from the pilot study were not included in the primary research. To evaluate the reliability of the questionnaire, Cronbach’s alpha was calculated and resulted in a value of 0.818, indicating a solid internal consistency. Data were collected using a self-administered online questionnaire in Arabic, which was specifically designed to assess parents’ knowledge and awareness regarding POSA across the Central Region through various social media channels such as Facebook, Telegram, and WhatsApp. The questionnaire had two parts: the first gathered socio-demographic details, and the second focused on assessing obstructive sleep apnea (OSA) knowledge. Participants responded to 14 questions in this section to gauge their awareness and understanding of POSA. Parents’ knowledge was measured by the number of correct answers related to the definition, symptoms, and risk factors of POSA. A cumulative knowledge score, with a maximum possible score of 30, was calculated by summing the weights of all correct answers. Participants were then grouped into two categories based on their scores: those with good knowledge (>70%) and those with poor knowledge (≤70%) [13].

Following data collection, the information was entered into Excel spreadsheets to clean and check for completeness, eliminating any potential errors or discrepancies. The data were then coded in preparation for analysis. All analyses were conducted using SPSS version 27. Descriptive statistics for categorical data were reported as frequencies and percentages. Chi-square tests were used to analyze categorical variables. Additionally, multivariate analysis was conducted to control for confounding variables like baseline BMI, duration of treatment, co-administration of other medications, presence of pre-existing gastrointestinal disorders, and lifestyle-related factors and to determine independent associations. The results were subsequently organized and presented in tables, graphs, and descriptive formats.

Ethical clearance was obtained from Qassim University’s ethical committee. All the participants involved in this study received informed consent along with the questionnaire, as participation was entirely voluntary. Participants could withdraw from the study at any time. Personal identifying information was not gathered during the study to maintain anonymity. All data collected remained confidential, with access limited to the principal investigator and the co-investigator.

## 3. Results

A total of 838 participants completed the online questionnaires (Table 1). A considerable proportion, 271 (32.4%) of them, were aged 31 to 40 years; more than half, 478 (57.0%), were females, and the majority, 803 (95.8%), were Saudi nationals. The distribution of respondents was evenly split between the Riyadh and Qassim regions, with each contributing 50% to the total sample size. Most, 757 (90.3%), of the parents were married; the majority, 510 (60.9%), had a bachelor’s education level, and nearly one third, 258 (30.8%), were teachers. Slightly more than half, 430 (51.3%) of them, had one to three children.

Table 2 (below) depicts the questions on knowledge regarding POSA. A substantial proportion, 261 (31.2%) of the parents, reported internet and social networking sites as their source of knowledge about POSA; more than half, 467 (55.7%), were aware of its correct definition as episodes of recurrent sleep apnea. Nearly one quarter, 221 (26.4%), of the participants reported knowing someone with OSA, and 236 (28.2%) of them attested to having a child who suffered from the condition. Around half, 425 (50.7%), correctly reported snoring as a common symptom of POSA. Risk factors that were correctly reported included mouth breathing (156, 18.6%), obesity (373, 44.5%), and enlarged tonsils (175, 20.9%). A notable proportion, 321 (38.3%), reported voluntary awareness campaigns as the most appropriate measure for raising public awareness about POSA.

Table 3 (below) shows the parents’ awareness of POSA and reveals insightful trends. The majority of the patients demonstrated a need for more general knowledge regarding POSA. Less than half of them knew of the effect of OSA on children’s academic performance, 351 (41.9%); its impact on children’s attention and behavior, 317 (37.8%); its association with depression, 251 (30.0%); and genetics as its risk factor, 418 (49.9%). On the other hand, the majority of them knew that pediatric OSA can be treated, 697 (83.2%); early treatment and diagnosis can reduce potential complications, 680 (81.1%); and parental awareness has a role in reducing the financial burden associated with OSA, 716 (85.4%).

Table 4 presents the relationship between parents’ demographic information and their knowledge level regarding POSA. Seven (7) questions were used to measure the parents’ knowledge. Each statement had two options: either “Yes” or “No”. Every correct answer was assigned a score of 1, while the incorrect answer was assigned 0. The highest score any participant could achieve was 7, and the lowest score any respondent could achieve was 0. Data were converted to composite scores. The level of knowledge was categorized as follows: those with good knowledge (>70%) had a score of 5–7 and those with poor knowledge (≤70%) had a score of 0–4. Data were then analyzed to find out the levels of knowledge among the parents.

Overall, 320 (38.2%) of the parents had good knowledge, while 518 (61.8%) exhibited poor knowledge about POSA. The result established a statistically significant association between age, gender, marital status, education level, specialization/work, source of knowledge about the problem of POSA, knowing someone with OSA, and having a child who suffers from OSA or snoring, and level of knowledge about POSA, with *p*-values of <0.001*. No statistically significant association existed between nationality, region, number of children, and knowledge about POSA (*p* > 0.05)**.** While the results are clearly presented, it is important to interpret them with caution due to the possibility of confounding variables. For instance, the observed gender difference in knowledge may not be solely attributable to gender itself but could be influenced by other factors such as educational attainment, caregiving roles, or exposure to healthcare information. Similarly, other demographic variables may be interrelated, potentially affecting the apparent strength of individual associations.

The results of the multiple logistic regression analysis (Table 5) identified several significant factors predicting poor parental awareness of pediatric obstructive sleep apnea (POSA). These factors include gender (AOR = 1.65; 95% CI = 1.220–2.223; *p* = 0.001), source of knowledge about pediatric obstructive sleep apnea (AOR = 1.35; 95% CI = 1.167–1.572; *p* < 0.001), and knowing someone with OSA (AOR = 1.92; 95% CI = 1.301–2.832; *p* = 0.001).

## 4. Discussion

POSA causes serious health problems in children, particularly during their growth and development [14]. The disorder is responsible for several children’s behavioral issues, which cause learning challenges, interfere with their academic performance, and lower their general quality of life [15]. If OSA is not recognized and treated, it can cause serious problems such as high blood pressure and metabolic and cardiovascular disease [16]. Parental knowledge of the signs, symptoms, and risk factors of POSA is critical for responding to early symptoms and selecting the best techniques for diagnosing and treating children with OSA. This study assessed the knowledge and awareness of parents in the Central Region regarding POSA.

The general knowledge among the parents in this region regarding POSA was found to be poor, with only 320 (38.2%) of the parents showing good awareness, while the majority, 518 (61.8%), demonstrated poor knowledge regarding the disorder. This poor level of knowledge is concerning given the potentially serious complications of untreated POSA, including behavioral issues, poor academic performance, and long-term cardiovascular or metabolic consequences. The findings are similar to those of a systematic review study conducted by McDowall et al. which reported poor parental knowledge regarding sleep disorders and their children’s quality of sleep [17].

Female parents demonstrated significantly better knowledge about pediatric OSA than male parents (*p* = 0.003*), which aligns with the recent study conducted in Jazan, Saudi Arabia, by Al-Makramani et al. who reported higher knowledge scores in female parents than males regarding pediatric OSA [13]. This gender-based difference may not be solely attributed to gender itself but could reflect broader social and cultural dynamics in Saudi Arabia. Women are often the primary caregivers in households and may be more involved in day-to-day childcare, medical appointments, and school-related matters. As a result, they may be more likely to notice early symptoms of sleep disorders and seek health-related information. The current study found that parents aged 41 to 50 years exhibited considerably better awareness regarding pediatric OSA than those in other age groups (*p* = 0.045*), contradicting the Al-Makramani et al. study, which did not find significant differences across the age groups. The discrepancy could be attributed to variations in the study population’s educational background, regional awareness efforts, or differences in sample size and recruitment methods. These variations underscore the importance of conducting further research with larger, more diverse populations to validate these findings and explore potential influencing factors [13].

The study found notable knowledge gaps among the parents, with the majority of those who had a middle level of education and lower demonstrating a significant lack of knowledge regarding pediatric OSA (*p* = 0.034*), which is consistent with the Owens et al. findings, which noted that parents who had lower levels of education exhibited poor knowledge regarding pediatric OSA [18]. The majority of parents who worked in the military had insufficient knowledge regarding pediatric OSA (*p* < 0.001*). Furthermore, parents who did not know a person with OSA demonstrated a considerable lack of general knowledge regarding pediatric OSA compared with other parents (*p* < 0.001*). This inadequate knowledge could potentially result in delayed pediatric OSA detection and treatment, causing long-term impacts on children’s health, cognitive development, and quality of life.

Parents had insufficient knowledge of the symptoms of pediatric obstructive sleep apnea, risk factors, its effects on children’s academic performance, and its association with mental health issues, many were unaware that signs such as mouth breathing could indicate OSA, and few recognized the role of genetic factors in its development. Additionally, there was limited awareness of the condition’s negative impact on children’s academic performance and its association with mental health issues, such as depression. The results corroborate those of Bokov et al. who found that mouth breathing and snoring in children with OSA are often disregarded or misinterpreted as normal juvenile behavior [19]. Nevertheless, these are underlying severe sleep disorders that require prompt treatment. The current study noted that parents of OSA-affected children had comparatively better parental knowledge regarding the illness than parents of children without such cases. A notable proportion, 261 (31.2%), of parents obtained information regarding pediatric OSA from the internet and social networking sites, highlighting the growing importance of digital health information and the potential for using online platforms to disseminate accurate and evidence-based information about pediatric OSA [20].

This study offers several strengths that contribute to the existing literature. First, it is one of the few studies conducted in the Central Region of Saudi Arabia that specifically evaluates parental awareness and knowledge of pediatric OSA, filling a significant research gap. Second, the study involved a relatively large and diverse sample, enhancing the reliability of the results. This study has limitations and drawbacks that must be addressed when assessing the results. The cross-sectional design could only determine the association between study attributes but not their causal relationships. The online format also introduces potential selection bias, as individuals who are more health-conscious or technologically literate may have been more likely to participate, potentially limiting the representativeness of the sample. Since the study relied on self-reported online survey data, recall and social desirability biases may have influenced its accuracy and dependability. Furthermore, because the study was limited to parents from Saudi Arabia’s Central Region, the findings cannot be generalized to other populations.

## 5. Conclusions

The general knowledge among the parents in the Central Region regarding pediatric obstructive sleep apnea was poor. The study noted significant knowledge gaps in identifying pediatric OSA symptoms and their effects on children’s mental health and academic performance, highlighting the need for implementing focused educational initiatives and programs for parents and healthcare providers to improve children’s mental well-being and overall health outcomes.

## Figures and Tables

**Table 1:** Socio-demographic information of the participants (n = 838).

Socio-Demographic Information	Category	Frequency and Proportion n (%)
Age	18 to 30	162 (19.3%)
	31 to 40	271 (32.4%)
	41 to 50	234 (27.9%)
	51 to 60	155 (18.5%)
	Older than 60	16 (1.9%)
Nationality	Saudi	803 (95.8%)
	Non-Saudi	35 (4.2%)
Region	Riyadh	419 (50.0%)
	Qassim	419 (50.0%)
Gender	Female	478 (57.0%)
	Male	360 (43.0%)
Marital status	Single	62 (7.4%)
	Married	757 (90.3%)
	Divorced	9 (1.1%)
	Widowed	10 (1.2%)
Education level	Uneducated	8 (1.0%)
	Secondary	131 (15.6%)
	Middle	33 (3.9%)
	Undergraduate university	80 (9.5%)
	Bachelor’s	510 (60.9%)
	Postgraduate studies	76 (9.1%)
Specialization/work	Healthcare worker	127 (15.2%)
	Engineer	56 (6.6%)
	Teacher	258 (30.8%)
	Pilot	13 (1.5%)
	Lawyer	29 (3.5%)
	Military	60 (7.2%)
	Private sector	24 (2.9%)
	Housewife	65 (7.8%)
	Student	13 (1.5%)
	Others	193 (23.0%)
Number of children	1 to 3	430 (51.3%)
	4 to 7	331 (39.5%)
	Eight and more	77 (9.2%)

Socio-demographic information presented in frequencies (n) and proportion (%).

**Table 2:** Knowledge about pediatric obstructive sleep apnea.

Questions	Categories	Frequency and Proportion n (%)
How did you know about the problem of obstructive sleep apnea in children?	A person with sleep apnea	182 (21.7%)
	Medical articles	111 (13.2%)
	Internet and social networking sites	261 (31.2%)
	I have never heard of it before	284 (33.9%)
What is pediatric obstructive sleep apnea?	A natural process that takes place while sleeping.	59 (7.0%)
	A total cessation of breathing that lasts from sleep onset until waking up.	42 (5.0%)
	Episodes of recurrent sleep apnea	467 (55.7%)
	I don’t know	270 (32.3%)
Is there someone you know who has faced this problem?	Yes	221 (26.4%)
	No	617 (73.6%)
Do you have a child who experiences sleep apnea or snores?	Yes	236 (28.2%)
	No	602 (71.8%)
If a child has obstructive sleep apnea, what are the expected symptoms?	Discomfort in sleeping	27 (3.2%)
	Excessive daytime sleepiness	18 (2.1%)
	Frequent coughing	9 (1.1%)
	Hyperactivity	13 (1.6%)
	Mouth breathing	156 (18.6%)
	Nightmares	6 (0.7%)
	Noticeable episodes of stopping breathing during sleep	20 (2.4%)
	Restless sleep	13 (1.6%)
	Snoring	425 (50.7%)
	I don’t know	151 (18.0%)
What do you believe are the risk factors for sleep apnea and snoring in children?	Asthma	48 (5.7%)
	Cerebral palsy	8 (1.0%)
	Diabetes	8 (1.0%)
	Diet	48 (5.7%)
	Down syndrome	3 (0.4%)
	Enlarged tonsils	175 (20.9%)
	Obesity	373 (44.5%)
	One or both parents suffer from sleep apnea and snoring	18 (2.1%)
	Sickle cell anemia	2 (0.2%)
	Sinus allergy	22 (2.6%)
	I don’t know	133 (15.9%)
What do you think is the best way to increase awareness about obstructive sleep apnea in children within society?	Consult a specialist doctor	299 (35.7%)
	Internet and social networking sites	218 (26.0%)
	Voluntary awareness campaigns	321 (38.3%)

Knowledge about POSA presented in frequencies (n) and proportion (%).

**Table 3:** Assessment of the parental awareness about POSA.

Questions	Category	n (%)
Are you aware that OSA in children can impact academic performance?	Yes	351 (41.9%)
	No	487 (58.1%)
Are you aware that children with OSA have a higher likelihood of experiencing depression than their peers?	Yes	251 (30.0%)
	No	587 (70.0%)
Are you aware that OSA in children can impact their attention and behavior?	Yes	317 (37.8%)
	No	521 (62.2%)
Do you believe that genetics contribute to the development of OSA in children?	Yes	418 (49.9%)
	No	420 (50.1%)
Do you think that genetics play a role in the development of OSA in children?	Yes	697 (83.2%)
	No	141 (16.8%)
Do you believe that early detection and treatment of POSA can reduce possible complications?	Yes	680 (81.1%)
	No	158 (18.9%)
Do you think that raising parental awareness about POSA can help reduce the burden on families and, in turn, on society as a whole?	Yes	716 (85.4%)
	No	122 (14.6%)

Assessment of the parental awareness about POSA presented in frequencies (n) and proportion (%).

**Table 4:** The association between socio-demographic information and parental awareness level about POSA.

Variables	Level of Knowledge			
	Category	Poor	Good	*p*-Value
Age	18 to 30	111 (68.5%)	51 (31.5%)	0.045 *
	31 to 40	159 (58.7%)	112 (41.3%)	
	41 to 50	132 (56.4%)	102 (43.6%)	
	51 to 60	105 (67.7%)	50 (32.3%)	
	Older than 60	11 (68.8%)	5 (31.3%)	
Nationality	Saudi	495 (61.6%)	308 (38.4%)	0.628
	Non-Saudi	23 (65.7%)	12 (34.3%)	
Region	Riyadh	268 (64.0%)	151 (36.0%)	0.201
	Qassim	250 (59.7%)	169 (40.3%)	
Gender	Female	275 (57.5%)	203 (42.5%)	0.003 *
	Male	243 (67.5%)	117 (32.5%)	
Marital status	Single	32 (51.6%)	30 (48.4%)	0.015 *
	Married	473 (62.5%)	284 (37.5%)	
	Divorced	9 (100.0%)	0	
	Widowed	4 (40.0%)	6 (60.0%)	
Education level	Uneducated	4 (50.0%)	4 (50.0%)	0.034 *
	Secondary	90 (68.7%)	41 (31.3%)	
	Middle	25 (75.8%)	8 (24.2%)	
	Undergraduate university	56 (70.0%)	24 (30.0%)	
	Bachelor’s	303 (59.4%)	207 (40.6%)	
	Postgraduate studies	40 (52.6%)	36 (47.4%)	
Specialization/work	Healthcare worker	78 (61.4%)	49 (38.6%)	<0.001 *
	Engineer	36 (64.3%)	20 (35.7%)	
	Teacher	134 (51.9%)	124 (48.1%)	
	Pilot	4 (30.8%)	9 (69.2%)	
	Lawyer	21 (72.4%)	8 (27.6%)	
	Military	49 (81.7%)	11 (18.3%)	
	Private sector	14 (58.3%)	10 (41.7%)	
	Housewife	39 (60.0%)	26 (40.0%)	
	Student	6 (46.2%)	7 (53.8%)	
	Others	137 (71.0%)	56 (29.0%)	
Number of children	1 to 3	261 (60.7%)	169 (39.3%)	0.523
	4 to 7	205 (61.9%)	126 (38.1%)	
	Eight and more	52 (67.5%)	25 (32.5%)	
Source of knowledge about POSA	A person with sleep apnea	90 (49.5%)	92 (50.5%)	<0.001 *
	Medical articles	64 (57.7%)	47 (42.3%)	
	Internet and social networking sites	138 (52.9%)	123 (47.1%)	
	I have never heard of it before	226 (79.6%)	58 (20.4%)	
Knowing someone with OSA	Yes	98 (44.3%)	123 (55.7%)	<0.001 *
	No	420 (68.1%)	197 (31.9%)	
Having a child who suffers from OSA or snoring	Yes	125 (53.0%)	111 (47.0%)	<0.001 *
	No	393 (65.3%)	209 (34.7%)	

Association between parents’ socio-demographic information and parental awareness level about pediatric obstructive sleep apnea. * Significant at *p* < 0.05 level.

**Table 5:** Factors predicting poor parental awareness of POSA.

Variables	AOR	95% CI	*p*-Value
Age	0.92	0.790–1.079	0.315
Nationality	1.29	0.611–2.720	0.504
Gender	1.65	1.220–2.223	0.001 *
Marital status	1.09	0.789–1.509	0.559
Education level	1.09	0.969–1.234	0.146
Specialization/work	1.04	0.993–1.088	0.098
Number of children	1.13	0.877–1.443	0.355
Source of knowledge about POSA	1.35	1.167–1.572	<0.001 *
Knowing someone with OSA	1.92	1.301–2.832	0.001 *
Having a child who suffers from OSA or snoring	1.09	0.762–1.546	0.651

AOR—adjusted odds ratio; CI—confidence interval. * Significant at *p* < 0.05 level.

## Data Availability

The data supporting this study are available upon reasonable request from the corresponding author.

## Abbreviations

The following abbreviations are used in this manuscript:

BMI	Body mass index
IRB	Institutional review board
KSA	Kingdom of Saudi Arabia
OSA	Obstructive sleep apnea
POSA	Pediatric obstructive sleep apnea
SPSS	Statistical Package for the Social Sciences
